# Supplementary material for: Fish evacuate smoothly respecting a social bubble
Source: Sci Rep. 2023 Jul 20;13:10414. doi: 10.1038/s41598-023-36869-9 (PMC10359245; doi:10.1038/s41598-023-36869-9)
Supplement: Supplementary file 1 — Supplementary Information. [file 41598_2023_36869_MOESM1_ESM.pdf]

Fish evacuate smoothly respecting a social bubble

Renaud Larrieu[1], Philippe Moreau[1], Christian Graff[2], Philippe Peyla[1], and Aurélie Dupont[1,*]

[1]Univ. Grenoble Alpes, CNRS, LIPhy, Grenoble, F-38000, France
[2]Univ. Grenoble Alpes, CNRS, LPNC, F-38000 Grenoble, France
[*]aurelie.dupont@univ-grenoble-alpes.fr

Contents

Figure S1: Number of fish evacuating simultaneously 2
Figure S2: Survival function of the time lapses 3
Figure S3: Complete evacuation dataset 4
Table S4: Summary table of the analyses 4
Supplementary notes: Statistics of egress in the case of independent events 5
Supplementary notes: Statistics of egress in the case of dependent events. 5

Figure S1:Number of fish evacuating simultaneously

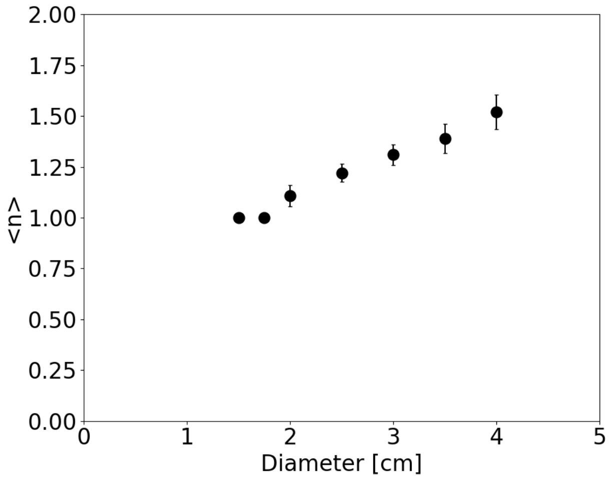

Figure S1: Average number of fish exiting between two images as a function of the opening diameter. The framerate of the image acquisition was 10 frame per second. For large openings, several fish could exit during this period of 0.1s, they are called simultaneous exits. This average number of fish exiting simultaneously is larger than one for diameters larger than 2cm, reaching 1.5 for the largest diameter of 4cm. Interestingly, <n> is strictly equal to one for the two smaller diameters. The fish do not evacuate side by side but have to strictly follow each other in what we called the dropper regime. Although their body width would allow it, fish do not line up according to body mechanical limits, unlike sheep for instance.

Figure S2: Survival function of the time lapses

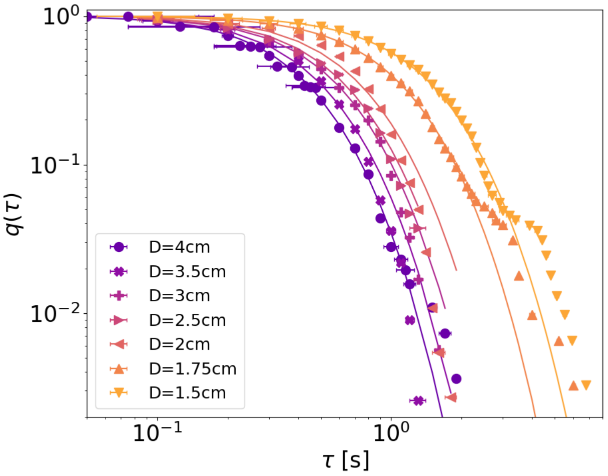

Figure S2: The complementary of the cumulative distribution function of the time lapses is shown in logarithmic scale on both axes. Hence, a power-law function would appear as a straight line. The fitted function overlays the data points for all diameters. No straight line is visible for the shorter time lapses as it is the case in other systems. One can notice a shouldering of the data from the two smallest diameters for time lapses about a couple of seconds. This concerns only a couple of points, it is therefore impossible to conclude to a power-law tail. In addition, the last points come back to the same trend of the fitting function.

Figure S3: Complete evacuation dataset

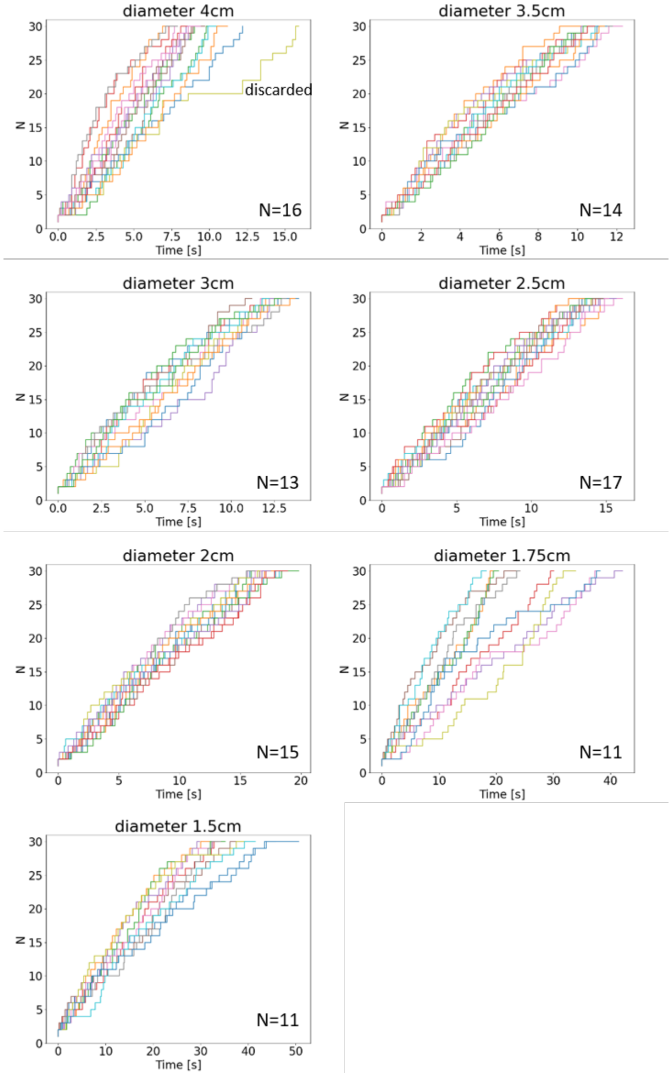

Figure S3: All the evacuation experimental dataset is shown for each diameter. Only one experiment was discarded in the case of an aperture of 4cm as a clear outlier.

Table S4: Summary table of the analyses

Diameter [cm]	Density [fish.cm^{-2}]	n [fish/event]	$<\Delta t>$ [s]	Fish current [fish.s^{-1}]	τ_0 [s]
1.5	0.30 ± 0.06	1.0	1.3 ± 0.4	0.95 ±0.05	0.777 ± 0.002
1.75	0.28 ±0.03	1.0	0.9 ± 0.2	1.2 ± 0.1	0.575 ± 0.001
2.0	0.23 ± 0.02	1.11 ± 0.05	0.6 ± 0.1	1.86 ± 0.05	0.385 ± 0.002
2.5	0.21 ± 0.02	1.22 ± 0.05	0.5 ± 0.1	2.12 ± 0.07	0.331 ± 0.001
3.0	0.22 ±0.03	1.31 ± 0.05	0.4± 0.1	2.5 ± 0.1	0.307 ± 0.001
3.5	0.21 ± 0.03	1.39 ± 0.07	0.38 ± 0.09	3.0 ± 0.1	0.263 ± 0.001
4.0	0.20 ± 0.03	1.52 ± 0.09	0.33 ± 0.1	3.3 ± 0.3	0.230 ± 0.001

Statistics of egress in the case of independent events

If we assume that exit events are independent, then the probability $q_i(\tau)$ that there is no event during the time τ, where i holds for "independent", and is such as:

$$q_i(\tau+\delta\tau) = q_i(\tau)q_i(\delta\tau), \tag{1}$$

where $q_i(\delta\tau) = 1-\delta\tau/\tau_0$, The time τ_0 being a characteristic time with $\delta\tau \ll \tau_0$. Equation (1) becomes:

$$q_i(\tau+\delta\tau) = q_i(\tau)\left(1-\delta\tau/\tau_0\right).$$

or

$$\frac{1}{q_i}\frac{\delta q_i}{\delta\tau} = -\frac{1}{\tau_0} \tag{2}$$

which reads

$$q_i(\tau) = \exp(-\tau/\tau_0). \tag{3}$$

with $q_i(\tau=0)=1$. Note that $q_i(\tau)$ is such as $q_i(\tau) = \int_\tau^{+\infty}\frac{1}{\tau_0}\exp(-\tau'/\tau_0)d\tau'$, with an event distribution that follows an exponential distribution $\rho(\tau) = \frac{1}{\tau_0}\exp(-\tau/\tau_0)$. However, $q_i(\tau)$ does not fit well our data.

Statistics of egress in the case of dependent events.

Now we assume that there is no statistical independence for short time scale. We want to include that each fish cannot exit immediately after its predecessor which exited before it. We can write that:

$$q(\tau+\delta\tau) = q(\tau)q(\delta\tau|\tau),$$

or

$$q(\tau+\delta\tau) = q(\tau)\left[1-p(\delta\tau|\tau)\right],$$

where $p(\delta\tau|\tau)$ is the probability of an exit knowing that no event occurs during a typical time τ_0. We propose $p(\delta\tau|\tau) = \frac{1-exp(-\tau/\tau_0)}{\tau_0}\delta\tau$

$$\frac{1}{q}\frac{\delta q}{\delta\tau} = -\frac{1-exp(-\tau/\tau_0)}{\tau_0}, \tag{4}$$

that can be compared with (2). If $t \gg \tau_0$ we recover the statistical independence, and $\frac{1}{q}\frac{\delta q}{\delta\tau} \approx -\frac{1}{\tau_0}$. But for short time scales, when $\tau \ll \tau_0$, then $\frac{1}{q}\frac{\delta q}{\delta\tau} \approx \frac{\tau}{\tau_0^2} \to 0$ when $t \to 0$. It means that if the time laps since the last exit is very recent, the next exit is not very probable. Thus, the function $\frac{1-exp(-\tau/\tau_0)}{\tau_0}$ interpolates the two regimes at long and short time scales . It gives:

$$q(\tau) = \exp\left[1-\frac{\tau}{\tau_0}-\exp\left(-\frac{\tau}{\tau_0}\right)\right]. \tag{5}$$
